# Mass spectrometry analysis of the photosystem II assembly factor Psb27 revealed variations in its lipid modification

**DOI:** 10.1007/s11120-021-00891-7

**Published:** 2021-12-15

**Authors:** Jan Lambertz, Pasqual Liauw, Julian P. Whitelegge, Marc M. Nowaczyk

**Affiliations:** 1 Plant Biochemistry, Faculty of Biology and Biotechnology, Ruhr-University Bochum, Bochum, Germany; 2 The Pasarow Mass Spectrometry Laboratory, David Geffen School of Medicine, The Jane and Terry Semel Institute for Neuroscience and Human Behavior, UCLA, Los Angeles, CA 90095 USA

**Keywords:** Photosynthesis, Psb27, Cyanobacteria, Lipoprotein, Mass spectrometry

## Abstract

The assembly of large, multi-cofactor membrane protein complexes like photosystem II (PSII) requires a high level of coordination. The process is facilitated by a large network of auxiliary proteins that bind transiently to unassembled subunits, preassembled modules or intermediate states of PSII, which are comprised of a subset of subunits. However, analysis of these immature, partially assembled PSII complexes is hampered by their low abundance and intrinsic instability. In this study, PSII was purified from the thermophilic cyanobacterium *Thermosynechococcus elongatus* via Twin-Strep-tagged CP43 and further separated by ion exchange chromatography into mature and immature complexes. Mass spectrometry analysis of the immature Psb27-PSII intermediate revealed six different Psb27 proteoforms with distinct lipid modifications. The maturation and functional role of thylakoid localized lipoproteins are discussed.

## Introduction

Photosystem II (PSII), the water-splitting enzyme of oxygenic photosynthesis, is a large membrane protein complex that consists of more than 20 subunits and many cofactors (Umena et al. 2011). Biogenesis of PSII and its repair in cyanobacteria involve a stepwise assembly process facilitated by numerous auxiliary protein factors, which are not part of the active, dimeric protein complex (Heinz et al. 2016; Komenda et al. 2012; Mabbitt et al. 2014). Psb27, one of these auxiliary factors, was identified in partially assembled PSII complexes of cyanobacteria (Kashino et al. 2002; Nowaczyk et al. 2006). It binds to an inactive PSII assembly intermediate, which is almost fully assembled except for binding of the extrinsic subunits (PsbO, PsbU and PsbV). Psb27 plays a role in PSII assembly as well as repair, particularly under stress conditions (Chen et al. 2006; Grasse et al. 2011; Roose and Pakrasi 2008). Two orthologues of Psb27 are present in *Arabidopsis thaliana* (At1g03600, At1g05385) and both are involved in PSII assembly or repair, with the latter (also referred to as LPA19) being more related to assembly (Chen et al. 2006; Wei et al. 2010). Although genes encoding similar proteins are also present in other photosynthetic eukaryotes like red and green algae, as well as diatoms, their function has not been studied yet.

The structure of isolated cyanobacterial and plant Psb27 has been solved four times in total, two times by NMR spectroscopy (Cormann et al. 2009; Mabbitt et al. 2009) and two times by X-ray crystallography (Michoux et al. 2012; Xingxing et al. 2018). All structures revealed a four-helix bundle with a right-handed up-down-up-down topology. The exact binding position of Psb27 on partially assembled PSII complexes and its structural function was a matter of debate for some time. Sequence analysis revealed the presence of a signal sequence that indicated a binding position at the PSII lumenal side, which was supported by in vitro reconstitution experiments (Nowaczyk et al. 2006). The Psb27 structural models enabled in silico docking (Cormann et al. 2009) as well as mass spectrometry (MS)-based methods for the identification of its binding position on PSII (Cormann et al. 2016; Liu et al. 2013, 2011), however with contradictory results (see also Heinz et al. (2016)). Very recently, two groups solved structures of Psb27-containing PSII complexes by cryo-electron microscopy, which show the Psb27 binding position on PSII with atomic resolution (Huang et al. 2021; Zabret et al. 2021). Psb27 is oriented with helix 2 and helix 3 towards PSII and forms specific contacts with the CP43 lumenal loop regions between helix 3 and helix 4, as well as with the E-loop between helix 5 and 6.

Association of Psb27 with free CP43 was shown previously based on biochemical data (Komenda et al. 2012) and its specific localization might indicate a general stabilizing function of Psb27 for the large lumenal E-loop of CP43 – e.g. chaperoning the association of free CP43 to the reaction center complex with CP47 (RC47)—and an indirect role for the assembly and photoactivation of the Mn_4_CaO_5_ cluster, the water-splitting center of PSII. Psb27 is – as most of the analyzed assembly factors – not essential for PSII biogenesis and repair, at least under optimal growth conditions (Grasse et al. 2011; Roose and Pakrasi 2008) and its binding does not induce obvious structural differences (Zabret et al. 2021). The structure of the CP43 E-loop is almost identical in all available structures – at least from thermophilic cyanobacteria—independent of the presence or absence of Psb27 or the extrinsic proteins (Zabret et al. 2021). However, in Spinach this domain seems to be more flexible without the extrinsic proteins according to a recent high-speed atomic force microscopy study (Tokano et al. 2020) and in the mesophilic cyanobacterium *Synechocystis* sp. PCC 6803 (in the following *Synechocystis*), binding of Psb27 supports PSII photoactivation, probably by stabilizing CP43 in a specific conformation (Avramov et al. 2020). Psb27 has only little overlap with the binding position of PsbO but its presence might still lower the binding affinity of PsbO, which would support the initial idea that Psb27 may prevent the premature binding of PsbO to keep the site of cluster assembly in a more open conformation for the incorporation of ions (Becker et al. 2011; Nowaczyk et al. 2006). Interestingly, a density was found in the Psb27-PSII structure, which might correspond to the first manganese atom bound to the high-affinity site in the process of PSII photoactivation (Zabret et al. 2021). Moreover, the D1 C-terminus, which is important for the coordination of the Mn_4_CaO_5_ cluster in mature PSII, was resolved and it clearly adopts a different conformation, which opens the cluster binding site (Zabret et al. 2021).

Another striking feature of cyanobacterial Psb27 is its N-terminal lipid modification. Psb27 is translated with an N-terminal signal sequence, which is cleaved off at a specific site during transfer through the thylakoid membrane. The mature protein starts with an N-terminal cysteine residue, which is modified by a thioether linked diacyl-glycerol moiety and a single acyl chain attached to the N-terminus (Nowaczyk et al. 2006). Although the Psb27 amino acid chain was traced to the N-terminal cysteine residue, the actual lipid modification is not visible in the current structure of the complex (Zabret et al. 2021).

In the present study, a novel method for the isolation of highly purified (intermediate) complexes from *T. elongatus* via TwinStrep-tagged PSII is described. This method enabled detailed mass spectrometry analysis of an intermediate Psb27-PSII complex, revealing six Psb27 proteoforms with different lipid modifications.

## Experimental procedures

### Generation and cultivation of *T. elongatus* strains

The PsbC-subunit (CP43) of the *Thermosynechococcus elongatus* (*T. elongatus*) BP-1 wild-type was extended at the C-terminus by a Twin-Strep(TS)-Tag (Schmidt et al. 2013) with the sequence GSSAWSHPQFEKGGGSGGGSGGSAWSPQFEK as described previously (Zabret et al. 2021). For the isolation of His-tagged PSII, the same strain and conditions were used as described before (Grasse et al. 2011; Nowaczyk et al. 2006). Cultures were cultivated in BG-11 medium at 45 °C in 25 L-photobioreactors (Bioengineering) supplied with 5% CO_2_. Light intensity was adjusted to cell density from 50 to 300 µmol photons·m^−2^·s^−1^ (Kuhl et al. 2000).

### Purification of photosystem II and sample preparation

Thylakoid membranes and proteins samples were prepared based on previous work (Kuhl et al. 2000; Nowaczyk et al. 2006; Zabret et al. 2021). In brief, isolated membranes were resuspended and homogenized in a Dounce homogenizer for three times in 80 ml Buffer B (20 mM Tris pH 7.5, 10 mM MgCl_2_, 10 mM CaCl_2_, 0.5 M mannitol). After second homogenization, 0.05% (w/v) n-dodecyl-β-D-maltoside (β-DDM) were added. After centrifugation (22,000 rcf, 15 min, 4 °C) in a JLA16.250 rotor, pellets were resuspended in extraction buffer (Buffer B supplied with 1.2% n-dodecyl β-D-maltoside, 0.5% (w/v) Na-chelate and a spatula tip DNase I) and the chlorophyll content was adjusted to 1 mg·ml^−1^. After incubation for 30 min at 20 °C, the sample was ultracentrifuged for 1 h at 4 °C (Beckmann Type 45 Ti Fixed-Angle Titanium Rotor, 102,800 rcf). The resulting supernatant was filtered and applied on the corresponding affinity chromatography columns. For the purification with the His-Tag, all buffers were used with 20 mM MES, pH 6.5 instead of Tris-Buffers. To prevent any photodamage, all steps were performed in the dark or under dim green light.

Streptactin affinity chromatography was performed with IBA Lifescienes streptactin Superflow hc cartridges (5 ml) in TS-Equilibration Buffer (Buffer B with 150 mM NaCl, 0.03% (w/v) β-DDM). Proteins were eluted in TS-Elution Buffer (TS-Equilibration Buffer supplied with 2.5 mM desthiobiotin). Immobilized Ni^2+^-Chelate affinity chromatography (IMAC) was performed as described before (Nowaczyk et al. 2006). After affinity chromatography, the elution buffers were exchanged to IEC-Equilibration Buffer (20 mM MES pH 6.5, 10 mM MgCl_2_, 10 mM CaCl_2_, 0.5 M mannitol, 0.03% (w/v) β-DDM) and proteins were further separated by anion exchange chromatography (IEC) (Kuhl et al. 2000). Samples were applied onto UNOQ6 columns (Bio-Rad) and eluted with IEC-Elution Buffer (IEC-Equilibration buffer with 100 mM MgSO_4_).

### Analysis of proteins with polyacrylamide gel electrophoresis

The oligomeric state of the protein complexes was determined with Blue Native PAGE (Schägger and Jagow 1991) with a gradient from 3.5 to 16% polyacrylamide. Proteins were separated by denaturing SDS-PAGE in an 12% polyacrylamide gel with 5 M urea (Schägger and Jagow 1987). As a size standard, PageRuler #26614 (Thermo Scientific) was used.

### Oxygen evolution measurements

Oxygen evolution was measured under continuous illumination with strong white light (Schott Fiber Optics™ KL 2500 LCD Illuminator, 1,300 lm, color temperature: 3,300 Kelvin) at 30 °C in a home-made setup using a Fibox 3 system with an oxygen sensitive optode (DP-PSt3-L2.5-ST10-YOP, PreSens Precision Sensing GmbH). The system was calibrated using air-bubbled (100% O_2_) and sodium dithionite saturated (0% O_2_) water.

PSII samples were diluted in 1 ml of activity buffer (20 mM MES pH 6.5, 1 M betaine, 10 mM CaCl_2_, 10 mM MgCl_2_, 0.03% (w/v) β-DDM) to a final concentration of 2–5 µg Chl·ml^−1^, supplied with 5 mM potassium ferricyanide and 1 mM 2,6-dichloro-1,4-benzoquinone (DCBQ) as artificial electron acceptors and incubated in the dark until a stable baseline was reached. After turning on the light, oxygen evolution was monitored for at least 20 s in the linear range.

### Mass spectrometry analysis

Samples were analyzed as described previously (Thangaraj et al. 2010). Purified proteins were precipitated in 80% acetone for 1 h at − 20 °C to remove salts, detergent and most pigments. Proteins were resuspended in 90% formic acid and the different subunits of the PSII complexes were separated at a flow rate of 100 µl·min^−1^ via reversed-phase liquid chromatography (RP-LC) by a PLRP-S column (particles: 5 µm, pores: 300 Å, 2·100 mm; Agilent) on a 140B Solvent Delivery System; Applied Biosystems. The columns were heated to 45 °C. Proteins were eluted by a discontinuous gradient from 0–90% (mobile phase: 0.05% TFA in 1:1 1-propanole/acetonitrile; stationary phase 0.05% TFA in distilled water) over 180 min. Eluates were split, one part analyzed by ESI–MS with a low-resolution mass spectrometer (LTQ, Thermo Finnigan) and the other collected in a fraction collector and later used for high-resolution top-down FTICR MS (7 T LTQ FT; Thermo Scientific). There, the total ion count (TIC) of the protein spectra was detected in a mass range of 700–2,000 m/z. Analysis of the spectra was done with MagTran (Version 1.03. (Zhang and Marshall 1998)). Fractions were stored at −80 °C until usage.

Top-down MS with selected fractions from the LC–MS runs was performed in a FTICR MS mass spectrometer (Thermo Scientific) with nanospray ionization at 1.7–1.9 kV in metal-steamed emitter tips (Proxeon) at a flow rate of 20–50 nl·min^−1^. TIC was analyzed at a mass range of 300–2,000 m/z with a resolution of 60,000 and isolated ions were separated via collision-induced dissociation (CID) at 15–20%. MS3 spectra were obtained for Psb27 via an additional ion isolation and CID. Obtained data were analyzed with ProSightPC (Version 2.0, Thermo Scientific) against a database of *T. elongatus* proteins.

## Results

**Fig. 1:**
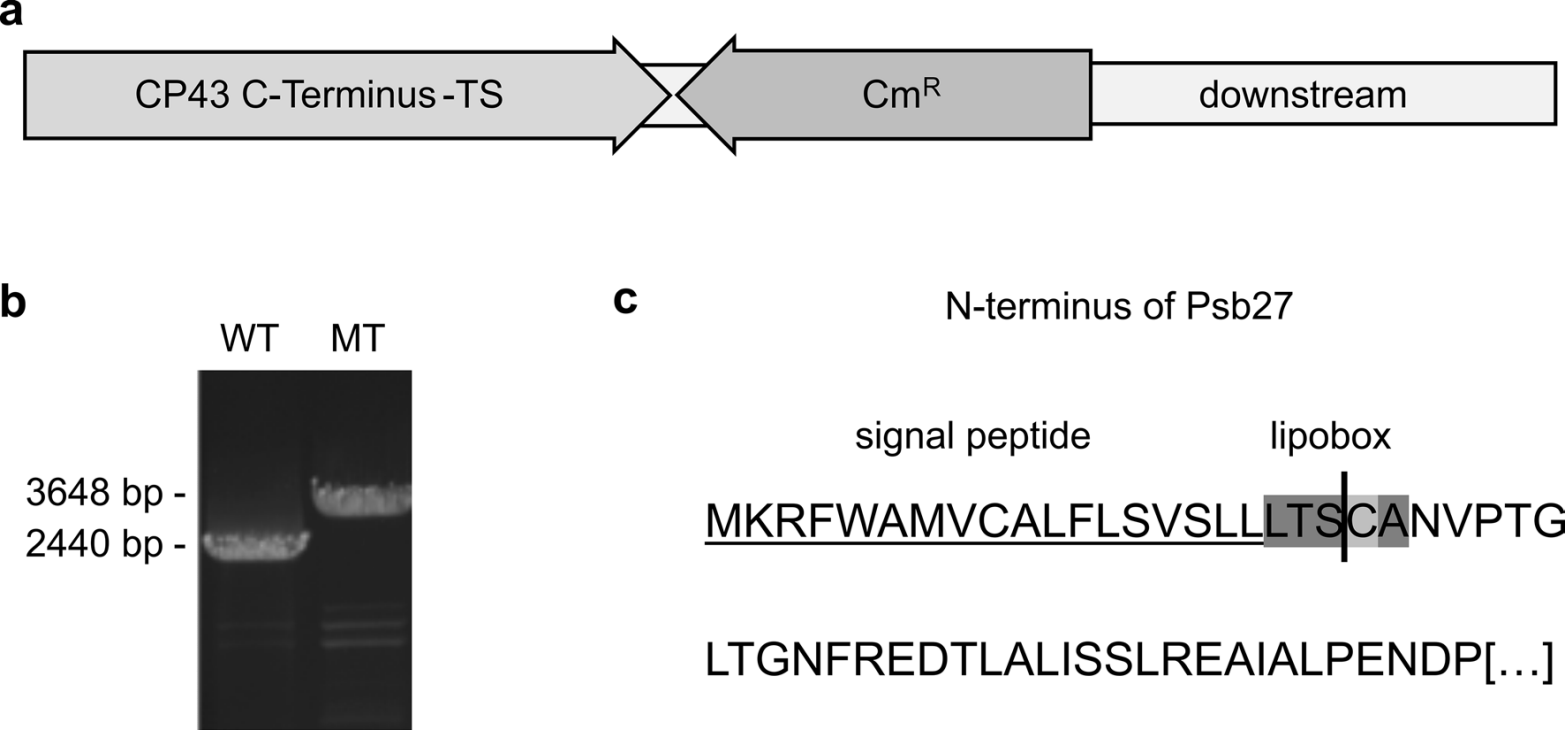
Construction of the CP43-TS-Tag mutant and amino acid sequence of the Psb27 N-terminus. **a** Schematic view of the construct that was used to generate the CP43-TS-Tag mutant (Cm^R^: chloramphenicol-resistance cassette) and **b** segregation analysis by PCR with genomic DNA from *T. elongatus* wild-type (WT) and mutant (MT) as template. **c** Amino acid sequence of Psb27 with its signal septide (underlined), the lipobox motif (grey) and the conserved N-terminal cysteine residue (light grey), which is modified by lipidation after cleavage of the signal peptide (consensus sequence of the lipobox: [LVI]^−3^[ASTVI]^−2^[GAS]^−1^[C]^1^). Cleavage occurs directly before the cysteine (vertical line)

**Fig. 2:**
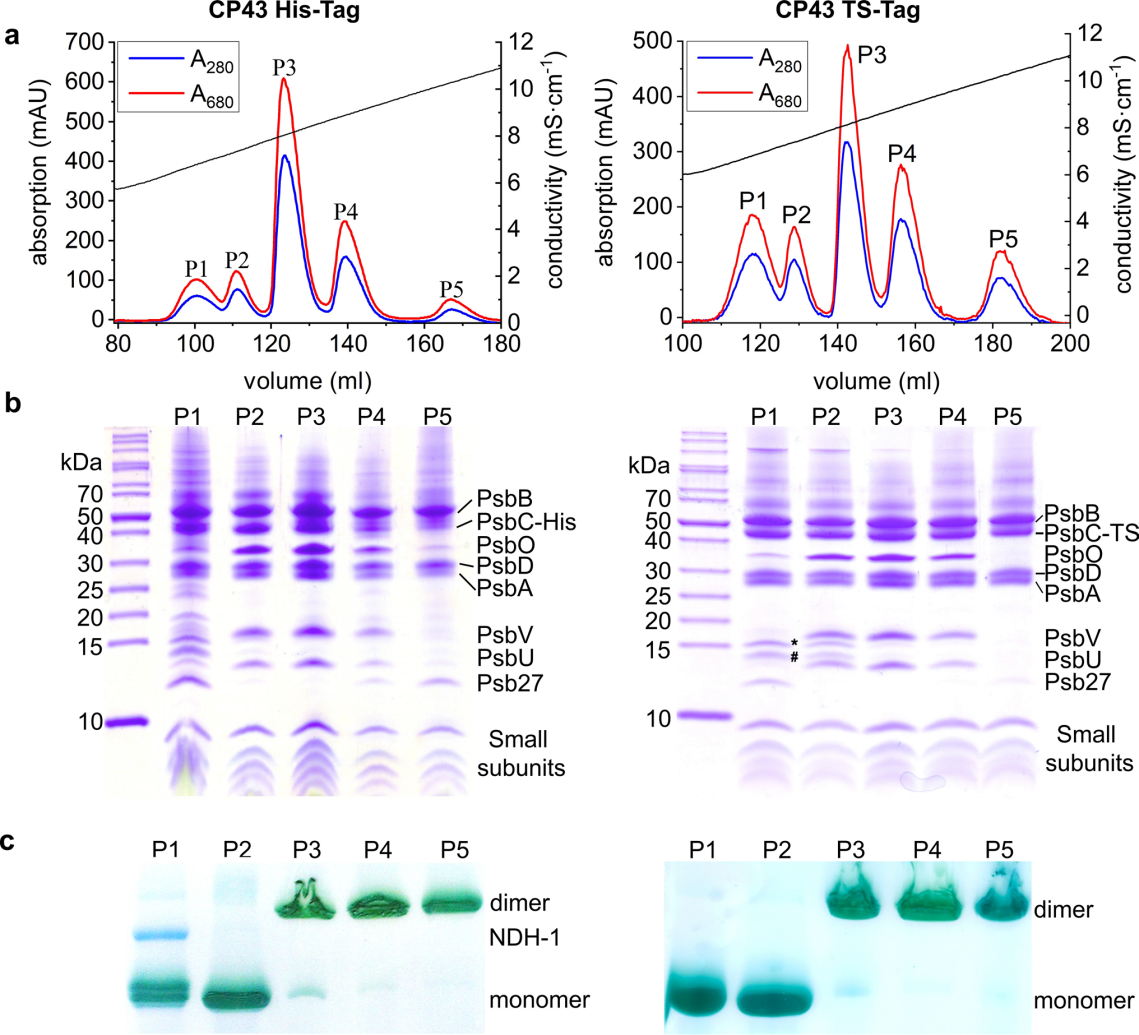
Purification of PSII complexes via tagged CP43 by IMAC chromatography (left) or purification with the TS-Tag (right). **a** IEC-Chromatograms of affinity purified PSII. **b** Subunit composition of the isolated PSII complexes by SDS-PAGE and **c** confirmation of mono- and dimeric states by BN-PAGE. * and # were assigned to phycobilisome subunits

A *T. elongatus* mutant line with a Twin-Strep-Tag (TS-Tag) fused to the PsbC (CP43) C-terminus was created (Fig. 1) and native PSII complexes of high purity were isolated by Strep-Tag affinity chromatography. Further separation of different (intermediate) PSII complexes was achieved by anion exchange chromatography (IEC). The results were compared with a previously established PSII preparation via His-tagged CP43 (Grasse et al. 2011; Nowaczyk et al. 2006) (Fig. 2a). With both techniques, two monomeric and three dimeric protein complexes were separated during IEC, showing the same, expected subunit composition (Fig. 2b and c, Table 1): All isolated complexes contain the core subunits D1, D2, CP43 and CP47, as well as several small subunits. The first IEC peak corresponds to monomeric PSII containing the auxiliary factor Psb27, whereas the extrinsic subunits PsbU and PsbV are missing. PsbO is not completely absent in this sample, most likely due to the overlap between the first and the second IEC peak fraction. The fractions 2–4 contain complexes with the protein composition of mature PSII. These complexes show oxygen evolution rates as reported before (Table 1), even though the pH was changed from 6.5 to 7.5 during affinity chromatography to comply with the requirements of the streptactin column material. The fifth fraction corresponds to a dimeric PSII complex with Psb27, but without the extrinsic subunits (Grasse et al. 2011). In comparison, the amount of co-isolated proteins is clearly reduced in the PSII TS-preparation. Particularly, the purity of the inactive, monomeric Psb27-PSII complex is increased by completely removing photosynthetic complex I (aka NDH-1), which is usually co-purified during IMAC purification of His-tagged PSII (Fig. 2). However, several subunits of the very high abundant cyanobacterial light harvesting complexes (phycobilisomes) are still present in this PSII fraction.

**Table 1:** Characteristics of isolated PSII complexes

IEC Peak	P1	P2	P3	P4	P5
Oligomeric state	Monomeric	Monomeric	Dimeric	Dimeric	Dimeric
Relative amount (%)	11	9	50	23	7
Activity (µmol O_2_·mg Chl^−1^·h^−1^)	112	2,814	4,900	2,120	0
Complex	Psb27-PSII	PSII_M(act)_	PSII_D(act)_	PSII_D(low)_	PSII_D(inac)_

PSII activities were determined by measuring the O_2_ evolution rates of isolated protein complexes

Due to the high purity of the Psb27-PSII complex, it was an ideal candidate for further characterization by top-down mass spectrometry to gain novel insights into post translational protein modifications, particularly into the lipid modification of Psb27. In contrast to a bottom-up approach, where proteins are cleaved into peptides by site-specific enzymes (e.g. trypsin), top-down MS is focused on intact proteins. The method is challenging, particularly for membrane proteins, as larger (and often hydrophobic) entities must be separated by RP-LC and subsequently analyzed by mass spectrometry. However, it enables precise assignment of post translational modifications based on the mass of the intact protein and it provides also information about the amino acid sequence, as peptide bonds within the intact protein can be cleaved by collision-induced dissociation (CID) in a subsequent MS/MS experiment.

**Fig. 3:**
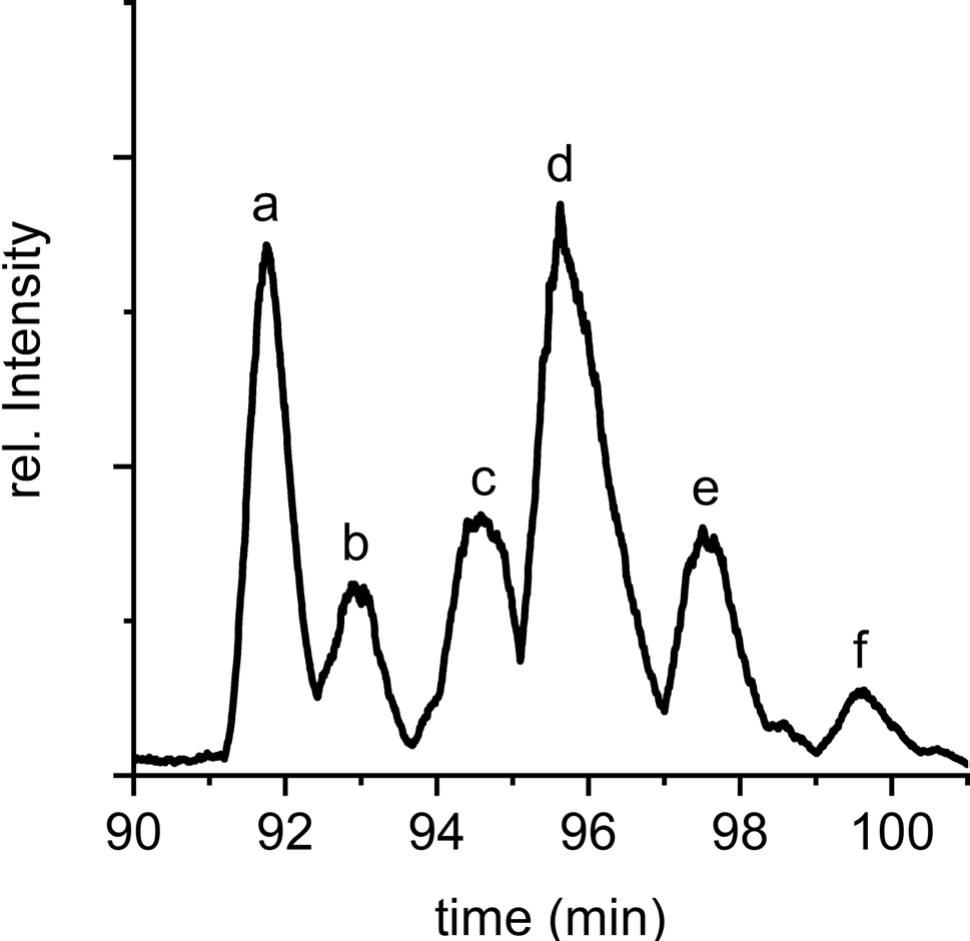
LC–MS TIC (basepeak) of Psb27 species a to f of inactive, monomeric PSII (Peak 1, PSII_(M,inac)_) after separation by RP-HPLC. The masses of the Psb27 species are shown in Table 1

**Fig. 4:**
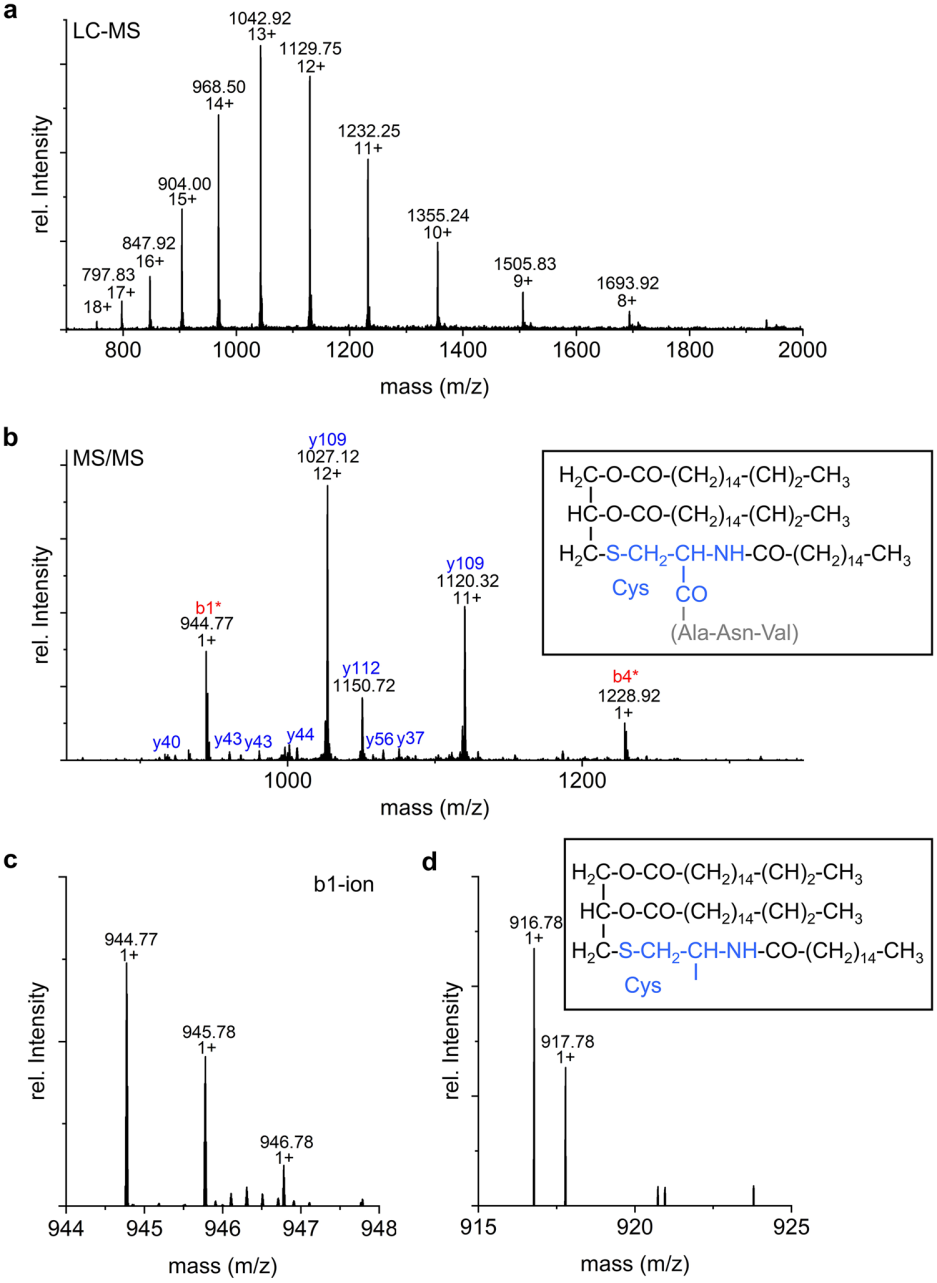
Top-down MS of Psb27 proteoform d. **a** Distribution of intact ions of Psb27d proteoform. **b** MS2-spectrum at 1,043.40 m/z after CID. b-ion series are labelled in red, y-ion series in blue. *indicates b-ions found after addition of the proposed modification at the N-terminus by a specific lipid modification with a mass of 842.76 Da. Box: Modification (black) of the cysteine (light blue) of the b1-ion. Brackets (grey): additional amino acids of the b4-ion. **c** Closer view of the b1-ion from **b** and its isotopic pattern. **d** MS3-spectrum of the Psb27d b1-ion at 945.00 m/z. The reduced mass (− 28 Da) results from the loss of the carboxy group of Psb27 by cleavage. The possible remaining a1-ion is shown on the top-right (box)

**Fig. 5:**
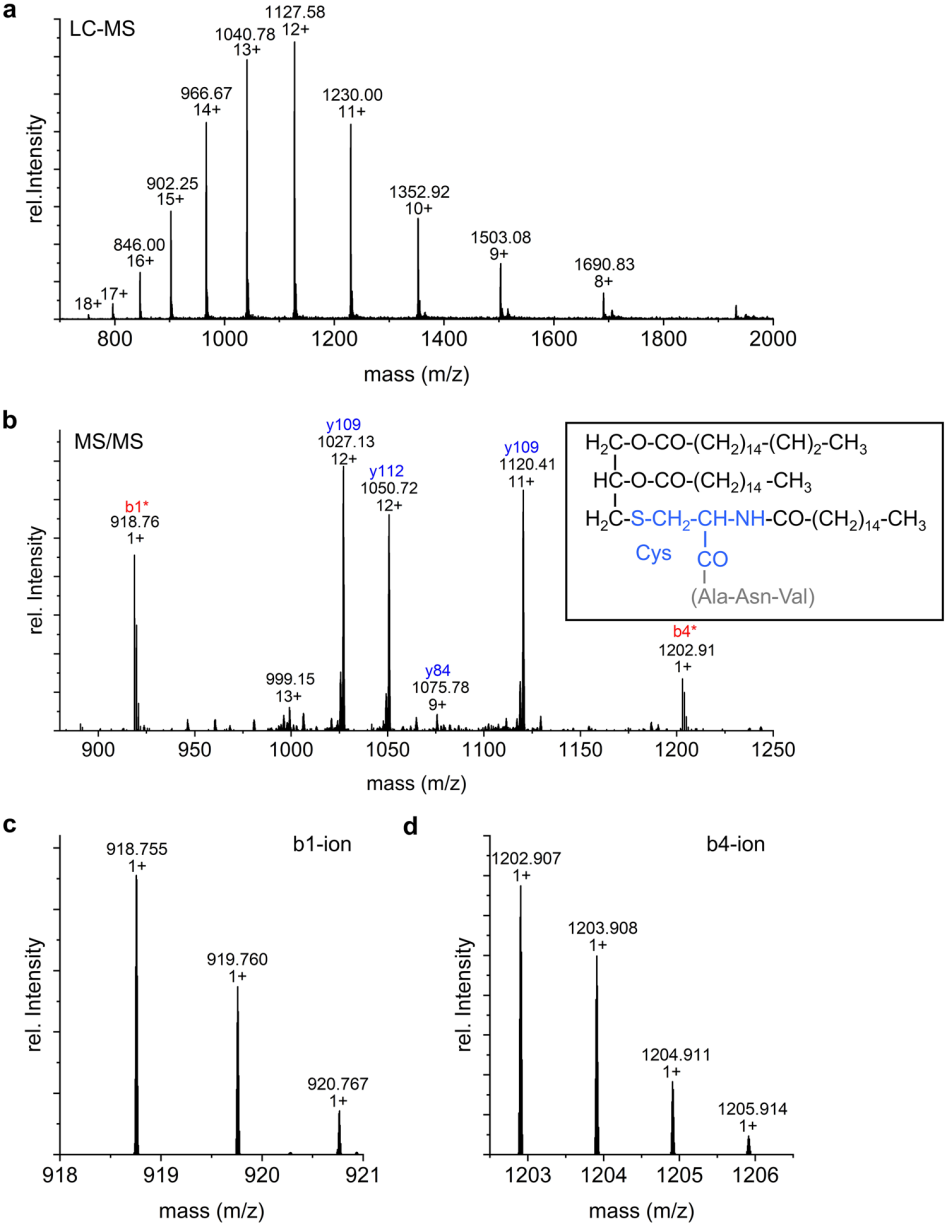
Top-down MS of Psb27 proteoforms. **a** Distribution of intact ions of Psb27c proteoform. **b** MS2-spectrum at 1,040.72 m/z after CID. b-ion series are labelled in red, y-ion series in blue. *indicates b-ions found after addition of the proposed modification at the N-terminus by a specific lipid modification with a mass of 814.76 Da (box). Box: Modification (black) of the cysteine (light blue) of the b1-ion. Brackets (grey): additional amino acids of the b4-ion. **c** Examples of assigned ions. Closer view of the b1-ion (left) and b4-ion (right) and their isotopic pattern

**Fig. 6:**
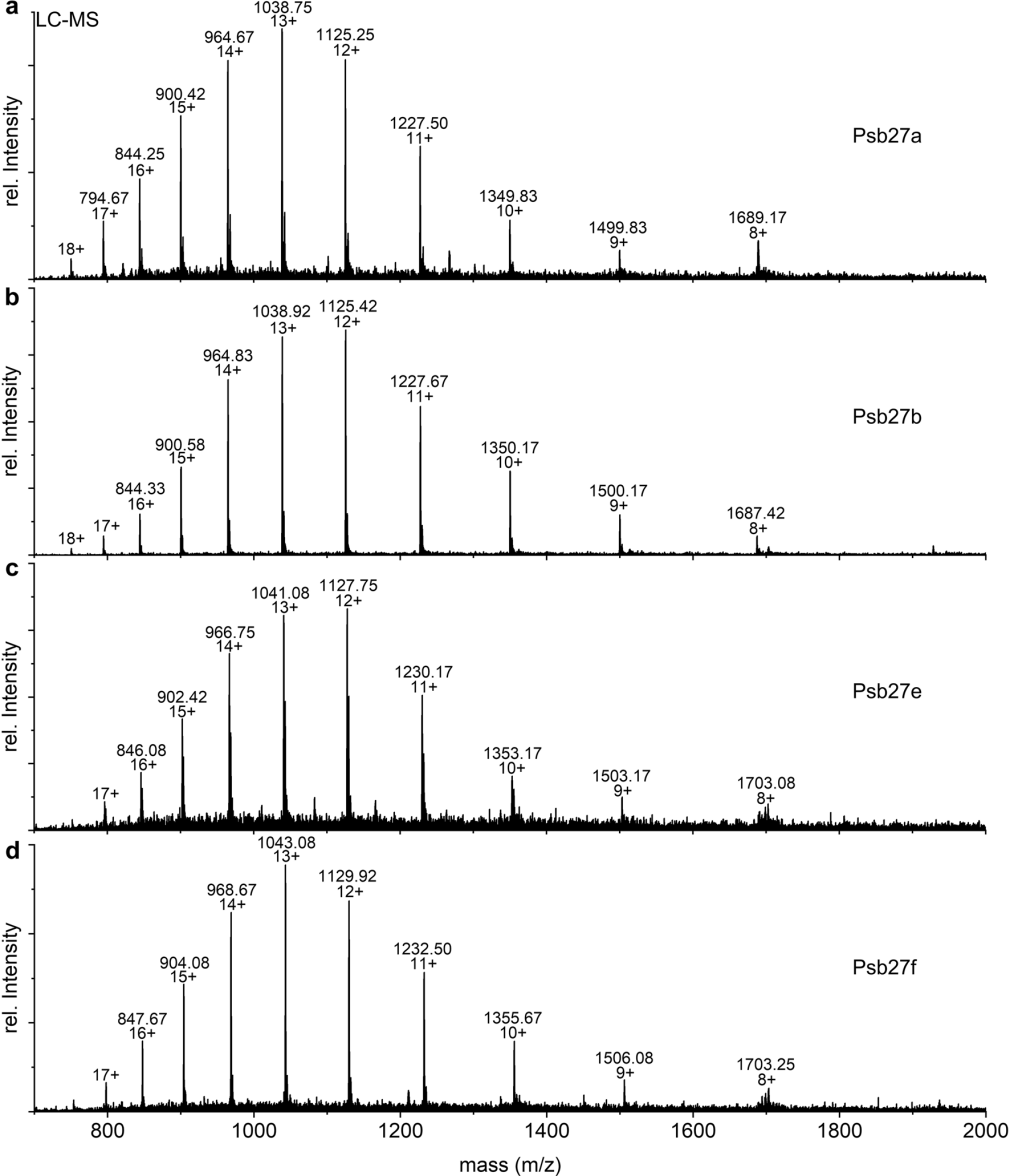
Distribution of intact ions from different Psb27 species acquired from LC–MS: Spectra of intact ions correspond to the elution peaks a, b, e and f in Fig. 3. Labels indicate the detected m/z and charge state of the ions

Consequently, proteins of the Psb27-PSII complex were separated in the first step by RP-LC and analyzed by MS to acquire low-resolution m/z values for each component. An aliquot of the eluates was collected, and specific fractions were used for subsequent analysis by high-resolution MS/MS. In the primary LC–MS dataset, Psb27 was found in six different proteoforms (a-f) with very similar retention times (Fig. 3) and masses (Fig. 4a, 5a and 6). The presence of Psb27 was confirmed by high-resolution MS/MS analysis of the different fractions (Fig. 4b and 5b), where initially only y-ions (those including the protein’s C-terminus), but no b-ions could be assigned to Psb27. The assignment of spectra is limited by unknown modifications due to the lack of knowledge about the exact position, mass and quantity. Based on previous data on Psb27 (Nowaczyk et al. 2006), the lack of N-terminal ions can be traced back to the lipid modification of the N-terminal cysteine of Psb27. The small mass differences between the Psb27 proteoforms indicate typical variations of the diacyl-glycerol moiety due to heterogeneity of the fatty acid composition (Fagerlund and Eaton-Rye 2011). The first ion at the N-terminus, called b1-ion, consists of only the (modified) cysteine and therefore is the most interesting ion to detect. To enable the assignment of b-ions, the total protein mass of the different Psb27 species was calculated from the m/z values acquired in the initial LC–MS experiment (Table 2, Fig. 4a, 5a and 6) and compared to the theoretical mass of the unmodified Psb27 sequence. Appropriate combinations of known lipids/fatty acids from cyanobacterial membranes (Allakhverdiev et al. 2001) were examined to explain the observed mass difference. Only by increasing the mass by 842.76 Da (C_55_O_5_H_102_), N-terminal b-ions could be assigned to species d in the MS/MS spectra (Fig. 4b, red ions). The detected y-ions (blue) remained unchanged. This mass corresponds to the substitution mass of a cysteine modification with glycerol, two oleic acids [C18:1] and one palmitic acid [C16:0] (Fig. 4b, box). By applying this modification, the b1-ion (944.773 m/z, Fig. 4c) and the b4-ion (C_mod_ANV, mass: 1,228.92 m/z) were identified, suggesting a modification of Psb27 species d by this lipid-combination. Other fatty acids did not yield any comparable cross-correlations. The b1-ion itself was further cleaved by CID to acquire MS3 spectra that resulted in a loss of 28 Da (Fig. 4d), which corresponds most likely to a dissociation of the carboxy group (Tabb et al. 2003), yielding the a1-ion, instead of further cleavage of peptide bonds.

**Table 2:** Lipid-modifications of the Psb27 proteoforms a to f

Species	RT (min)	MW (Da)	Modification	Possible fatty acids
a	92.86	13,491	C_51_O_5_H_96_	C14:1, C16:0, C16:1
b	94.58	13,493	C_51_O_5_H_98_	C14:1, 2 C16:0
c	95.62	13,519	C_53_O_5_H_100_	2 C16:0, C18:1
d	97.50	13,545	C_55_O_5_H_102_	C16:0, 2 C18:1
e	98.56	13,521	C_53_O_5_H_102_	2 C16:0, C18:0
f	99.63	13,547	C_55_O_5_H_104_	C16:0, C18:0, C18:1

Possible fatty acid combinations of the proteoforms are summarized. *RT* retention time, *MW* molecular weight

Similar as for species d, only y-ions were detected for Psb27 species c (Fig. 5b, blue). The N-terminal b-ions of the Psb27 species c, including the b1- (C_mod_) and b4-ion (Fig. 5c and d, respectively), were successfully assigned after addition of C_53_O_5_H_100_ (817.35 Da), which corresponds to a modification of the N-terminal cysteine with two C16:0 and one C18:1 fatty acid (Fig. 5b, box).

Based on the successful identification of the lipids of Psb27 species c and d, the modifications of the remaining species a, b, e and f were assigned by comparing the mass differences of the detected intact ions (Fig. 6a–d) with the theoretical mass of Psb27. Only specific combinations of the masses of stearic acid (C18:0), oleic acid (C18:1), palmitic acid (C16:0) and palmitoleic acid (C16:1), together with the diacyl-glycerol moiety, fit to the calculated mass differences and were therefore assigned as modifications of the Psb27 N-terminus (Table 2). The presence of five different fatty acids reveals a high variability of the Psb27 lipid modification. However, our data does not distinguish, which fatty acid residue is at the N-terminal amine, or the sn1 or sn2 position of the glycerol sidechain residue.

## Discussion

Achieving high protein purity is one important goal in protein biochemistry, as all further steps of characterization depend on sample quality. Integrity of the sample and its homogeneity are for example important criteria for structural or spectroscopic investigations. Large, multi-subunit membrane protein complexes, like PSII, are particularly challenging to isolate due to their high complexity and hydrophobic nature. Moreover, PSII assembly intermediates are even more challenging to isolate due to their low abundance and intrinsic instability, as reviewed in Heinz et al. (2016). Therefore, highly specific affinity purification methods are of great importance for these approaches.

Here, we compared the isolation of PSII complexes from *T. elongatus* by two different affinity chromatography methods: immobilized metal affinity chromatography with His-tagged CP43 and streptactin affinity chromatography by use of Strep-tagged CP43, respectively. Based on our previous experience with Strep-tagged membrane protein complexes (Schuller et al. 2019, 2020) we selected the sequence of two Strep-Tag II affinity markers (WSHPQFEK) separated by a spacer sequence (GGGSGGSGGSA). This Double- or Twin-Strep-Tag (TS-Tag) increases the affinity towards the streptactin affinity matrix, as it binds to two of the four Strep-Tag binding sites within the streptactin tetramer (Schmidt et al. 2013). The affinity purification step was followed by IEC to separate five different PSII species, which differ in protein composition and oligomeric state (Grasse et al. 2011). The first IEC fraction represents a PSII assembly intermediate with bound Psb27 and particularly the purity of this fraction was improved by use of the TS-Tag/streptactin system. Photosynthetic complex I, which is the main contaminant of Psb27-PSII isolated via CP43-His from *T. elongatus* is absent if CP43-TS-Tag is used. Moreover, the TS-Tag has no negative effect on the growth of the culture, the oxygen evolution rate or the stability of the isolated complex. The isolated Psb27-PSII complex was further analyzed by top-down mass spectrometry to investigate the Psb27 lipid modification in detail.

An estimated amount of 1%–3% of all bacterial genes encode lipoproteins and for *T. elongatus*, 0.44%–0.81% of all proteins are predicted lipoproteins (Babu et al. 2006). They are targeted to lipidation by the lipobox sequence motive, which is part of the cleavage site of an N-terminal signal peptide (Fig. 1c). After cleavage of the signal sequence by signal peptidase II during transport through the membrane, the conserved N-terminal cysteine residue is modified by diacyl-glyceryl transferase with the diacylglycerol moiety of a phosphatidyl glycerol (PG) molecule (Gan et al. 1993). Finally, the processed N-terminus is additionally acylated with a single acyl chain by N-acyl transferase (Tokunaga et al. 1982).

The role of lipoproteins in cyanobacteria may be underestimated because detailed knowledge about their structure and function is often missing. In *Syncechocystis*, at least 40 lipoproteins have been identified, and at least four of these are related to photosynthesis, as reviewed by Wada and Murata (2007). However, the precise function of the lipid modification itself is still elusive. Proteins might be tethered to the membrane just to limit diffusion in a two-dimensional space, which can be also mediated by a transmembrane helix (e.g. evolved from a former signal peptide) or the lipid moiety might play an additional role in the localization of the protein in a specific membrane or part of a membrane (Ferguson 1991; Okuda and Tokuda 2011; Juneau et al. 2016). For photosynthesis associated CyanoQ, the lipidation is essential for processing and accumulation of the protein in the thylakoids, independent of the signal peptide itself, which emphasizes the importance of the process (Juneau et al. 2016).

In general, the observed variance of the Psb27 lipid modification is typical for cyanobacterial lipoproteins since these proteins tend to bind lipids with different fatty acids (Fagerlund and Eaton-Rye 2011; Knoppová et al. 2021; Nowaczyk et al. 2006; Ujihara et al. 2008), which originate from membrane phospholipids (Chattopadhyay and Wu 1977; Lai and Wu 1980). Therefore, the type of lipid modification is determined by the fatty acid composition of the membrane, which in turn changes depending on environmental conditions (Allakhverdiev et al. 2001; Gombos et al. 1997; Mironov et al. 2012).

Cyanobacterial lipoproteins have been reported both in thylakoid and cytoplasmic membranes (Liberton et al. 2016), but in contrast to the Sec-pathway itself (Liberton et al. 2016; Nakai et al. 1993; Srivastava et al. 2005), a maturation pathway for lipidation was not yet identified in cyanobacterial thylakoid membranes (Agarwal et al. 2010; Liberton et al. 2016), apart from low abundant apolipoprotein N-acyltransferase. If the maturation system is not present in the thylakoid membrane, Psb27 and other photosynthesis related lipoproteins must be transferred efficiently after lipidation to the thylakoid membrane, as they are absent or only in low abundance in the plasma membrane (Ishikawa et al. 2005; Knoppová et al. 2021; Selão et al. 2016). Alternatively, lipoproteins might be specifically modified in the PratA-defined membrane (PDM) region at the interface between plasma and thylakoid membrane (Rast et al. 2016) and the transfer could be mediated by maturation centers, where both membranes are supposed to interact (Rast et al. 2019; Stengel et al. 2012). Interestingly, lipidation of Ycf48, another auxiliary protein involved in early PSII assembly, occurs during or after its association with the D1 precursor protein (Knoppová et al. 2021; Zak et al. 2001), most likely within the PDM (Komenda et al. 2008; Rast et al. 2016), which would further support a localization of the lipidation machinery in the PDM.

A more complex role of the lipidation might be indicated by a recent study, which investigated the effect of externally supplied free fatty acids on cyanobacterial photosynthesis (Jimbo et al. 2020). The addition of 16:0 and 18:0 fatty acids, which are important components of the lipid modification as shown here for Psb27, increased protein – and particularly D1 – synthesis, as well as the rate of PSII repair (Jimbo et al. 2020). In contrast, addition of polyunsaturated fatty acids like 18:3 had an opposite effect (Jimbo et al. 2020) or hampered overall growth (Berlepsch et al. 2012). These effects might be, at least partially, related to the altered function or deregulation of photosynthetic lipoproteins.

Detailed investigations of the protein network involved in PSII assembly and repair in the past several years have led to comprehensive knowledge of the spatial–temporal organization of the process. Particularly, technical developments in the field of structural biology in combination with molecular dynamics simulations are now the main driver for a molecular-mechanistic understanding of the assembly process and the involved assembly factors. However, integration of the lipid or small molecule sphere is still missing due to experimental limitations and further studies are needed to investigate the protein-lipid interplay and its role in PSII assembly.

## Abbreviations

Chl: Chlorophyll
CID: Collision-induced dissociation
IEC: Ion-exchange chromatography
IMAC: Immobilized metal affinity chromatography
MS: Mass spectrometry
PDM: PratA-defined membrane
PG: Phosphatidyl glycerol
RP-LC: Reversed-phase liquid chromatography
PSII: Photosystem II
TIC: Total ion count
TS: Twin-Strep
